# Novel 5-HT_7_ receptor antagonists modulate intestinal immune responses and reduce severity of colitis

**DOI:** 10.1152/ajpgi.00299.2023

**Published:** 2024-05-07

**Authors:** Yun Han Kwon, Benjamin E. Blass, Huaqing Wang, Jensine A. Grondin, Suhrid Banskota, Kenneth Korzekwa, Min Ye, John C. Gordon, Dennis Colussi, Kevin M. Blattner, Daniel J. Canney, Waliul I. Khan

**Affiliations:** ^1^Farncombe Family Digestive Health Research Institute, McMaster University, Hamilton, Ontario, Canada; ^2^Department of Pathology and Molecular Medicine, McMaster University, Hamilton, Ontario, Canada; ^3^Department of Pharmaceuticals Sciences, Moulder Center for Drug Discovery Research, Temple University School of Pharmacy, Philadelphia, Pennsylvania, United States

**Keywords:** 5-HT, 5-HT_7_ receptor, 5-HT_7_ receptor antagonist, inflammatory bowel disease, serotonin

## Abstract

Inflammatory bowel disease (IBD) encompasses several debilitating chronic gastrointestinal (GI) inflammatory disorders, including Crohn’s disease and ulcerative colitis. In both conditions, mucosal inflammation is a key clinical presentation associated with altered serotonin (5-hydroxytryptamine or 5-HT) signaling. This altered 5-HT signaling is also found across various animal models of colitis. Of the 14 known receptor subtypes, 5-HT receptor type 7 (5-HT_7_) is one of the most recently discovered. We previously reported that blocking 5-HT signaling with either a selective 5-HT_7_ receptor antagonist (SB-269970) or genetic ablation alleviated intestinal inflammation in murine experimental models of colitis. Here, we developed novel antagonists, namely, MC-170073 and MC-230078, which target 5-HT_7_ receptors with high selectivity. We also investigated the in vivo efficacy of these antagonists in experimental colitis by using dextran sulfate sodium (DSS) and the transfer of CD4^+^CD45RB^high^ T cells to induce intestinal inflammation. Inhibition of 5-HT_7_ receptor signaling with the antagonists, MC-170073 and MC-230078, ameliorated intestinal inflammation in both acute and chronic colitis models, which was accompanied by lower histopathological damage and diminished levels of proinflammatory cytokines compared with vehicle-treated controls. Together, the data reveal that the pharmacological inhibition of 5-HT_7_ receptors by these selective antagonists ameliorates the severity of colitis across various experimental models and may, in the future, serve as a potential treatment option for patients with IBD. In addition, these findings support that 5-HT_7_ is a viable therapeutic target for IBD.

**NEW & NOTEWORTHY** This study demonstrates that the novel highly selective 5-HT_7_ receptor antagonists, MC-170073 and MC-230078, significantly alleviated the severity of colitis across models of experimental colitis. These findings suggest that inhibition of 5-HT_7_ receptor signaling by these new antagonists may serve as an alternative mode of treatment to diminish symptomology in those with inflammatory bowel disease.

## Graphical abstract

**Figure:**
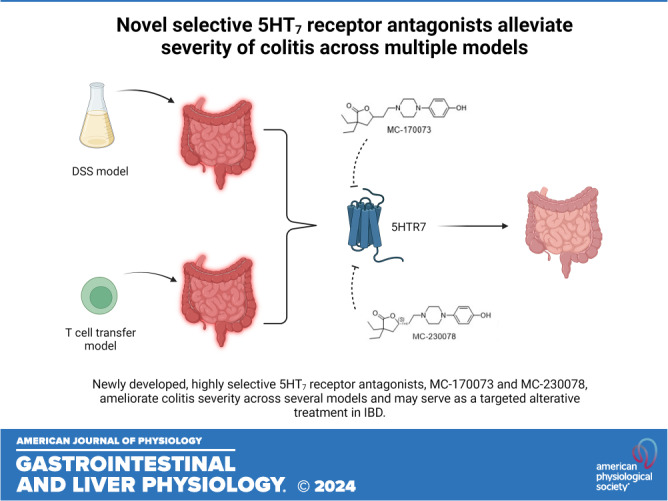

## INTRODUCTION

Inflammatory bowel diseases (IBDs), Crohn’s disease (CD), and ulcerative colitis (UC) are serious chronic inflammatory conditions of the human bowel, affecting over 3 million people in the United States and Canada and several million more worldwide (1, 2). Although death due to IBD is uncommon, the severe, chronic inflammation of the gastrointestinal tract associated with these conditions can produce significant complications that drastically impact patients’ lives and well-being. These include, but are not limited to, chronic pain, persistent diarrhea, rectal bleeding, fistula formation, intestinal strictures, abscesses, and the potential for bowel perforation. Thus, it is not surprising that IBD reduces the quality of life, decreases work capacity, and increases disability in individuals with these conditions. The burden that IBD places on individuals, the healthcare system, and society is significant (2, 3). Despite decades of research, there is no cure for IBD, and treatment options, at present, are limited. The current IBD standard of care focuses on disease management and symptom mitigation to minimize complications and decrease patient suffering. Symptomatic relief may be provided by anti-inflammatory medications such as aminosalicylates (e.g., 5-ASA) in mild-to-moderate cases. More severe cases can be treated with corticosteroids (e.g., prednisone), but long-term therapy with these drugs is discouraged due to severe side effects caused by extended treatment. Common long-term symptom suppression therapies include immunomodulators (e.g., 6-mercaptopurine, methotrexate, cyclosporin A) and anti-TNF-α biologics (4). However, the risk of toxicity in the case of immunosuppressive agents, acute infusion reactions, and possibly loss of efficacy over time are all substantial downsides associated with these drugs (4, 5).

Of note, symptom management using current standard-of-care medications is often insufficient. According to a 2022 study conducted by Khoudari et al. (6), up to 80% of patients with CD require surgery due to disease progression and complications associated with their condition. However, in CD, surgical removal of the afflicted intestinal region is not curative, with 50–60% of patients requiring additional surgery during their lifetime as a result of the reoccurrence of symptoms in a different region of the intestine (7). Surgical intervention in patients with UC is less frequent, though still significant, with ∼30% of patients requiring surgical intervention (6). Thus, it is imperative to investigate novel therapies that may minimize patient suffering and help avoid costly and undesirable surgical interventions.

For several decades, the development of novel therapeutics capable of slowing or reversing IBD progression has been a focus of pharmaceutical industries and academic laboratories alike. Evidence from both clinical and animal studies suggests that altered serotonin (5-hydroxytryptamine or 5-HT) production and signaling are present in intestinal inflammation and that 5-HT plays a distinct immunomodulatory role in the gut (8). These immunomodulatory effects are largely mediated through interactions with at least one of the 14 known 5-HT receptor subtypes_._ The most recent addition to the 5-HT receptor family, the 5-HT_7_ receptor, was first identified in 1993 (9). Of note, it is one of the five receptor subtypes found within the gut and is expressed on several immune cells (8). As part of the effort to develop a better understanding of IBD progression and develop new therapeutic approaches, we have previously investigated the role that this receptor plays in the context of colitis. Our work illustrated that 5-HT_7_ receptor-deficient (5-HT_7_^−/−^) mice are significantly less susceptible to dextran sodium sulfate (DSS)-induced colitis when compared with wild-type (WT) mice (10). In addition, we reported the impact of the highly selective 5-HT_7_ receptor antagonist, SB-269970 (*K*_i_ = 1.25 nM, EC_50_ = 1.05 nM) (11), in an acute model of DSS-induced colitis. Treatment with this compound significantly reduced the severity of colitis and the production of proinflammatory cytokines when compared with controls (10). Similar ameliorative effects have also been observed with SB-269970 treatment in dinitrobenzene sulfonic acid (DNBS)-induced colitis, though only a minor impact has been reported in 2,4,6-trinitrobenzenesulfonic acid (TNBS)-induced colitis (10, 12). Recent work has also demonstrated an upregulation of 5-HT_7_ receptor expression in mucosal samples from patients with CD as compared with healthy controls, suggesting altered 5-HT_7_ signaling is present in the clinical manifestation of IBD (13).

The aforementioned work strongly implicates 5-HT_7_ activation as a key component in the pathogenesis of colitis and has led to its identification as a new potential target to treat IBD. Here, in an effort to further explore the role of 5-HT_7_ in the pathogenesis of colitis, we conducted a series of studies investigating the impact of the novel 5-HT_7_ receptor antagonists, MC-170073 and MC-230078, in both the DSS and CD4^+^ T-cell transfer models of colitis (14). Our findings illustrate that blocking 5-HT_7_ receptor signaling with these novel antagonists significantly downregulated the production of proinflammatory cytokines and reduced the severity of intestinal inflammation across multiple models of colitis.

## MATERIALS AND METHODS

### Reagents

MC-170073, MC-20078, and MC-230079 were prepared as described in WO2014164756 (14).

### In Vitro Studies

#### 5-HT_7_ binding assay.

Solutions of test compounds were a 10 mM stock in dimethyl sulfoxide (DMSO) and then further diluted 1:200 in assay buffer [50 mM tris(hydroxymethyl)aminomethane (TRIS), pH 7.5, 10 mM MgCl_2_, 0.5 mM ethylenediaminetetraacetic acid (EDTA)] to 50 µM (0.5% DMSO). Ten dilutions of the test compound in 0.5% DMSO/assay buffer were prepared in a twofold serial dilution to yield final compound concentrations ranging from 10 µM to 19.5 nM in the assay. In a 96-well polypropylene plate, 50 µL of assay buffer was added to all wells, and then 50 µL of 0.5% DMSO/assay buffer or 50 µM chlorpromazine or serial dilutions of test compounds were added to selected wells. To all wells, 100 µL of 0.2 mg/mL human 5HT_7_A-HEK membranes and 50 µL of 5 nM [^3^H]lysergic acid diethylamide (LSD) in assay buffer were added. Reactions were incubated at room temperature for 1.5 h and harvested by rapid filtration onto 0.3% polyethyleneimine-treated GF/B filter plates using a Brandel harvester. Four rapid 500-μL washes were performed using chilled 50 mM TRIS buffer, pH 7.5. Filter plates were dried overnight, scintillant was added, and the radioactivity retained on the filters was counted on a TopCount scintillation counter (PerkinElmer). Half maximal inhibitory concentration (IC_50_) and inhibitory constant (*K*_i_) of test compounds were calculated using GraphPad’s Prism 5 nonlinear sigmoidal curve-fitting program. Specific binding equals total binding minus nonspecific binding. Nonspecific binding is defined by 10 µM chlorpromazine. *K*_i_ values were calculated using the Cheng-Prusoff equation.

#### 5-HT_7_ functional assays.

The potencies of MC-170073, MC-20078, and MC-230079 as 5-HT_7_ antagonists were determined using the cAMP Hunter eXpress GPCR luminescence assay (DiscoveRx, 95-0163E3) and the manufacturer’s protocol. Cryopreserved U2OS cells expressing h5-HT_7D_ were seeded into 96-well white-walled clear bottom tissue culture-treated plates (31,250 cells/well) and were allowed to recover for 16 h at 37°C in a humidified incubator with 5% CO_2_. The media was aspirated and replaced with 30-µL cell assay buffer (CAB). Cells were preincubated at 37°C for 15 min with 7.5 µL of 6× final concentration of 5 to 10 concentrations in triplicate of test compounds in CAB. Cells were then stimulated for 30 min at 37°C with 7.5 µL of 900 nM of the full agonist 5-HT in cell assay buffer (final concentration 150 nM; an EC_80_). cAMP levels/well were determined at room temperature by adding 15 µL of cAMP antibody and 60 µL of cAMP working detection solution, followed by the addition of 60 µL of solution A after 1 h incubation. Luminescence was read 16 h later with an EnVision multilabel plate reader. The IC_50_ of test compounds was calculated using GraphPad’s Prism 5 (GraphPad Software, La Jolla, CA) nonlinear curve-fitting program. The equilibrium dissociation constant for the competitive antagonists (*K*_B_) values was determined using the Cheng-Prusoff equation.

#### Off-target assessment.

In vitro assessments of MC-170073, MC-20078, and MC-230079 activity at the following targets were determined using the assay systems available through the Psychoactive Drug Screening Program (PDSP). Detailed protocols for each assay are available at https://pdsp.unc.edu/pdspweb/?site=assays. Receptors 5-HT_1A_, 5-HT_1B_, 5-HT_1D_, 5-HT_1E_, 5-HT_2A_, 5-HT_2B_, 5-HT_2C_, 5-HT_3_, 5-HT_5A_, 5-HT_6_, 5-HT_7_, α_1A_, α_1B_, α_1D_, α_2A_, α_2B_, α_2C_, benzodiazepine, β_1_, β_2_, β_3_, GABA-A, H_1_, H_2_, H_3_, H_4_, M_1_, M_2_, M_3_, M_4_, M_5_, δ-opioid, κ-opioid, and µ-opioid and transporters dopamine, norepinephrine, and serotonin were evaluated.

### Computational Values

Topological polar surface area (TPSA) and partition coefficient (cLogP) values were calculated using the Dotmatics software suite (Dotmatics, LLC, Bishop's Stortford, UK).

### Aqueous Solubility (pH 7.4) Assay

Compounds were assessed for their solubility at pH 7.4 using the commercially available Millipore MultiScreen Solubility filter system (Millipore, Billerica, MA). Analysis was performed by liquid chromatography-tandem mass spectrometry (LC-MS/MS).

### Cytochrome P450 Inhibition Assays

Compounds were assessed for their ability to inhibit human cytochrome P450 3A4 using testosterone as a substrate and LC-MS/MS analysis, whereas the 2D6 and 2C9 assays used fluorescent substrates and EnVision plate reader analysis. Expressed enzymes were used to minimize nonspecific binding and membrane partitioning issues (15).

### Microsomal Stability Assays

Test compounds were assessed for microsomal stability by incubating at 37°C in the presence of mouse or human liver microsomes and an NADPH regenerating system as described by Yang et al. (16). Microsomal protein content was adjusted to ensure accurate rates of substrate consumption. Analysis was performed by LC-MS/MS analysis.

### Mouse In Vivo Pharmacokinetic Studies

Adult C57BL/6 male mice (2–4 mo old, body weight ranging from 20 to 25 g, Charles River Laboratories) were maintained under controlled temperature (23 ± 1°C) and lighting (lights on 6:00 AM–6:00 PM) with food and water provided ad libitum. Pharmacokinetic studies were performed either via intravenous bolus infusion or through oral gavage. Mice were administered each compound intravenously by tail vein injection (1 mg/kg) or orally through gavage (5 mg/kg). Serial blood samples were collected at 5, 15, and 30 min and 1, 2, 4, 8, and 24 h after intravenous administration, and at 15, 30 min, and 1, 2, 4, 8, and 24 h after oral gavage administration. All blood samples were centrifuged at 1,500 *g* for 5 min. Pharmacokinetic study procedures were approved by Temple University’s Institutional Animal Care and Usage Committee and were in accordance with National Institutes of Health (NIH) guidelines. For all in vivo efficacy studies, experiments were approved by the animal ethics committee of McMaster University and conducted under Canadian guidelines for animal research.

### Plasma Protein Binding

Plasma protein binding was performed by using equilibrium dialysis (ED) (17). ED was performed using a 96-well equilibrium dialyzer with a molecular weight (MW) cutoff of 5 K (Harvard Apparatus, Holliston, MA) and placed in a dual-plate rotator set to maximum speed (Harvard Apparatus, Holliston, MA) located in a 37°C incubator with a 10% CO_2_ environment. After 22 h of dialysis, buffer and plasma were removed from each side of the dialysis plate. Samples were mixed with equal volumes of the opposite matrix (plasma or buffer) and stored at −20°C for future analyte quantitation.

### LC-MS/MS Analysis (PK and Plasma Protein Binding)

Plasma concentrations were determined using an LC-MS/MS method. Briefly, 100-µL acetonitrile with an internal standard solution (Diltiazem, 0.1 μg/mL) was added to 50-µL plasma or tissue homogenate. After being vortexed for 1 min, the mixture was centrifuged at 3,000 *g* for 5 min. LC-MS/MS analysis was carried out using a high-performance liquid chromatography system consisting of an Agilent 1100 system interfaced to an API4000 SCIEX triple-quadrupole tandem mass spectrometer (Applied Biosystems/MDS Sciex, Foster City, CA). MC-170073, MC-230078, MC-230079, and the internal standard were separated by using a Phenomenex (Torrance, CA) C18 column (2.0 × 50 mm). The mobile phase consisted of solvent A (0.1% formic acid in water) and solvent B (0.1% formic acid in acetonitrile). A linear gradient of solvent B from 5 to 95% was used. The detection and quantification of analytes were performed with the *m*/*z* transition 347/191 for MC-170073, MC-230078, and MC-230079, and *m*/*z* 415/178 for diltiazem.

### Mouse In Vivo Efficacy Studies

C57BL/6 (Taconic, Cambridge City, IN) for the DSS model of colitis, as well as C57BL/6 and Rag1^−/−^ mice (Jackson Laboratory, Bar Harbor, ME) for the T-cell transfer model, were kept in sterilized, filter-topped cages under specific pathogen-free conditions upon arrival and fed autoclaved food. Male mice aged 8–10 wk were used for all experiments. Mice were acclimatized for 7 days before the start of any experiments. All experiments were approved by the animal ethics committee of McMaster University and conducted under Canadian guidelines for animal research.

### DSS-Induced Colitis Model

For induction of acute colitis, DSS (molecular mass 40 kDa; ICN; Biomedicals Incorporate, Solon, OH) was added to autoclaved drinking water at a final concentration of 4 or 5% wt/vol for 5 days. For the induction of chronic colitis, DSS was added to normal drinking water at a final concentration of 4% for 5 days, followed by 7 days of water. This cycle was repeated twice thereafter, with 2% DSS. Control mice received vehicle or normal drinking water.

### T Cell-Induced Chronic Colitis Model

Colitis was induced in *Rag1^−/−^* mice by adoptive transfer of fluorescence-activated cell sorting (FACS)-sorted CD4^+^CD45RB^high^ T cells, as described previously (18). Briefly, CD4^+^ T cells were isolated from splenocytes of healthy C57BL/6 mice by the EasySep Mouse Naive CD4^+^ T Cell Isolation Kit (StemCell Technology, Vancouver, Canada). Naive CD4^+^ T cells were then labeled with PE-cy7-conjugated anti-mouse CD3 (BioLegend, San Diego, CA), allophycocyanin (APC)-conjugated anti-mouse CD4 (BD Biosciences), and fluorescein isothiocyanate (FITC)-conjugated anti-mouse CD45RB (BD Biosciences, Mississauga, Canada). CD4^+^CD45RB^high^ T cells were sorted using a FACS Aria II flow cytometer (BD Biosciences, Mississauga, Canada). Cell viability was assessed using a Trypan blue assay before injection. Recipients were administered ∼5 × 10^5^ sorted T cells via intraperitoneal injection.

### Drug Administration

For acute DSS-induced colitis, C576BL/6 mice were treated with MC-170073 (10 mg/kg of body wt) or vehicle (10% DMSO in normal saline) via intraperitoneal injection once daily for 6 days, starting 1 day before the beginning of DSS. In a separate experiment, C57BL/6 mice were orally administered with MC-230078 (10 mg/kg) or vehicle twice daily for 5 days, starting from the beginning of DSS. For chronic DSS-induced colitis, C57BL/6 mice were treated with MC-170073 (10 mg/kg ip) or vehicle once daily for 6 days, starting 1 day before the beginning of the third cycle. Last, for CD4^+^CD45RB^high^ T cell-induced chronic colitis, reconstituted Rag1^−/−^ mice were orally administered MC-230078 (5 mg/kg or 10 mg/kg) or a vehicle twice daily for 10 days starting from week 6 post-reconstitution of T cells.

### Disease Activity Index

Disease activity index (DAI) is a combined score of weight loss, stool consistency, and fecal bleeding and was blindly assessed using a previously published scoring system (19). This scoring system was defined as follows: weight loss: 0 = no loss; 1 = 1–5%; 2 = 5–10%; 3 = 10–20%; 4 = 20%+; stool, 0 = normal; 2 = loose stool; 4 = diarrhea; and bleeding: 0 = no blood; 2 = hemoccult positive (Hemoccult II; Beckman Coulter, Fullerton, CA); and 4 = gross blood (blood around anus). DAI was measured across all days of DSS treatment. Macroscopic damage scores were blindly scored using a previously published scoring system for DSS-induced colitis (19).

### Colonic Myeloperoxidase Level

Colonic myeloperoxidase (MPO) levels were measured following a published protocol (19). Briefly, colonic tissue samples were homogenized in ice-cold 50 mmol·L^−1^ potassium phosphate buffer containing 0.5% hexadecyl trimethyl ammonium bromide (pH = 6.0) (Sigma). Homogenates were centrifuged, the supernatant was removed, and an aliquot was then added to a solution containing potassium phosphate buffer, O-dianisidine, and hydrogen peroxide (Sigma-Aldrich). The absorbance was measured at 450 nm by a spectrophotometer (model EL808, BioTek). MPO level was expressed in units per milligram of tissue, where 1 U is defined as the quantity of enzyme able to convert 1 μmol hydrogen peroxide to water per minute at room temperature.

### Histological Analysis

Segments of the proximal colon were washed with PBS, fixed in 10% neutral-buffered formalin, washed with ethanol, and embedded in paraffin. Hematoxylin-eosin (H&E)-stained colonic tissue sections were scored using a previously published system (19): crypt architecture (normal = 0; severe crypt distortion with loss of entire crypts = 3), degree of inflammatory cell infiltration (normal = 0; dense inflammatory infiltrate = 3), muscle thickening (base of crypt sits on the muscularis mucosae = 0; marked muscle thickening present = 3), goblet cell depletion (absent = 0; present = 1), and crypt abscess (absent = 0; present = 1). A breakdown of scoring criteria can be seen in Supplemental Table S1. The histological damage score is the sum of each individual score. Images were captured using a Nikon Eclipse 80i microscope and NIS-Elements Basic Research imaging software. Investigators were blinded to the study groups.

### Enzyme-Linked Immunosorbent Assay

Colonic 5-HT content was determined as previously described (20). Briefly, a small section of the colon was weighed and homogenized in 0.2 N perchloric acid. After centrifugation at 10,000 *g* for 5 min, the supernatants were collected, and the pH was neutralized by using 1 mol·L^−1^ borate buffer. The supernatants were used for the analysis of 5-HT levels using a commercially available enzyme-linked immunosorbent assay (ELISA) kit (Cat. No. IM1749; Beckman Coulter, Fullerton, CA). 5-HT content is expressed as a function of tissue weight (ng·mg^−1^·tissue). For the determination of colonic cytokines, a small section of colonic tissue was homogenized in Tris-buffered saline containing a protease inhibitor mixture (Cat. No. P8340; Sigma-Aldrich, Oakville, Canada). Samples were centrifuged for 5 min at 3,300 *g*, and the resulting supernatants were frozen at −80°C until use. Total protein levels were quantified in the colon homogenates by using the DC Protein Assay Kit (Cat. No. 5000111; Bio-Rad Laboratories, Mississauga, Canada). Cytokine levels (IL-1β, Cat. No. SMLB00C; IL-6, Cat. No. SM6000B; IL-17A, Cat. No. M1700; IFN-γ, Cat. No. MIF00; and TNF-α, Cat. No. MTA00B) were determined according to the manufacturer’s instructions (Quantikine Murine; R&D Systems, Minneapolis, MN).

## RESULTS

### Compound MC-170073 Is a Potent and Highly Selective 5-HT_7_ Antagonist

**Figure 1.**
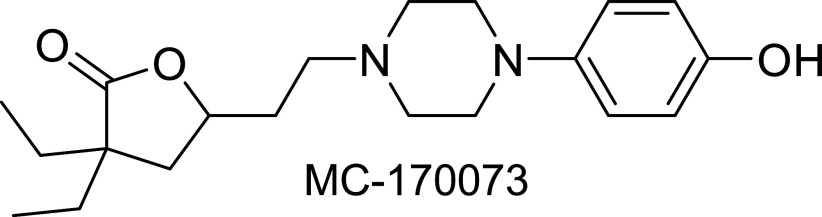
Structure of MC-170073.

As part of our ongoing effort to identify novel, selective 5-HT_7_ ligands and understand the role of this receptor in colitis, we explored a series of functionalized γ-butyrolactones. This effort led to the identification of MC-170073 (Fig. 1) as a potent 5-HT_7_ ligand (*K*_i_ = 89 nM) that acts as an antagonist at this receptor (*K*_b_ = 18 nM). An examination of the physicochemical properties (Table 1) of this compound revealed that it is highly soluble in aqueous media (200 µM), has a moderate half-life in mouse liver microsomes (*T*_1/2_ = 17 min), is highly stable in the presence of human liver microsomes (*T*_1/2_ > 60 min), and has limited capacity to inhibit the key metabolic enzymes CYP3A4, 2C9, and 2D6 (IC_50_ > 10,000 nM). Selectivity profiling at the remaining 5-HT receptors (5-HT_1A_, 5-HT_1B_, 5-HT_1D_, 5-HT_1E_, 5-HT_2A_, 5-HT_2B_, 5-HT_2C_, 5-HT_3_, 5-HT_4_, 5-HT_5A_, and 5-HT_6_) demonstrated that MC-170073 is a poor ligand for these receptors (*K*_i_ >10,000 nM). Additional selectivity profiling conducted through the National Institute of Mental Health’s (NIMH’s) Psychoactive Drug Screening Program (PDSP) revealed that this compound has limited binding capacity (*K*_i_ > 10,000 nM) at a variety of receptors and transporters, including α_1a_, α_1b_, α_1d_, β_1_, β_2_, β_3_, D_2_, D_5_, DAT, GABA-A, H_2,_ H_3_, H_4_, mAChR_1_, mAChR_2_, mAChR_3_, mAChR_4_, mAChR_5_, the opioid receptors (δ, κ, and µ), the benzodiazepine receptors (peripheral and brain), the norepinephrine transporter, and the serotonin transporter. Moderate activity was observed at α_2A_ (*K*_i_ = 3479 nM), α_2B_ (*K*_i_ = 493 nM), α_2C_ (*K*_i_ = 3,601 nM), D_1_ (*K*_i_ = 301 nM), D_3_ (*K*_i_ = 1,234 nM), D_4_ (*K*_i_ = 591 nM), and H_1_ (*K*_i_ = 915 nM).

**Table 1.** In vitro binding and physicochemical properties of MC-170073

MW	TPSA	cLogP	*K*_i_ for 5-HT_7_, nM	*K*_b_ for 5-HT_7_, nM	*K*_i_ for Other 5-HT Receptors, nM	IC_50_, nM			Sol, µM	*T*_1/2_, min	
						CYP3A4	CYP2C9	CYP2D6		MLM	HLM
346	53	3.1	89	18	>10,000	10,000	10,000	10,000	168	17	60

Calculated properties were determined using the Dotmatics software suite. 5-HT_7_ binding and functional data were generated using a [^3^H]LSD displacement assay and a cAMP production assay, respectively. Off-target 5-HT receptor activity was determined using assay services provided by the psychoactive drug screening service. Cyp inhibition was determined using enzyme inhibition assays. Solubility at pH 7.4 was determined using the commercially available Millipore MultiScreen solubility filter system. Microsomal stability was determined by incubating MC-170073 at 37°C in the presence of mouse or human liver microsomes and an NADPH regenerating system. 5-HT receptors include the following: 5-HT1A, 5-HT1B, 5-HT1D, 5-HT1E, 5-HT2A, 5-HT2B, 5-HT2C, 5-HT3, 5-HT4, 5-HT5A, and 5-HT6. cLogP, partition coefficient; HLM, human liver microsomes; IC_50_, half-maximal inhibitory concentration; *K*_b_, antagonist affinity constant; *K*_i_, dissociation constant; MLM, mouse liver microsome; MW, molecular weight; Sol, solubility; TPSA, topological polar surface area.

To determine the in vivo pharmacokinetic properties (noncompartmental analysis) of MC-170073, adult C57BL/6 male mice were administered a 1 mg/kg dose intravenously or a 5 mg/kg dose via oral gavage (Table 2). In both cases, MC-170073 demonstrated a moderate half-life (*T*_1/2_ = 4.4 and 3.4 h, respectively) and clearance (CL = 0.125 and 0.73 L/h, respectively). A comparison of the exposures produced in these experiments indicated that this compound has an oral bioavailability of 17%. Furthermore, these experiments illustrated that MC-170073 has a moderate capacity to bind to plasma protein (40%). Collectively, these findings suggest that MC-170073 is capable of reaching the systemic concentrations necessary to test its ability to elicit a pharmacological response.

**Table 2.** MC-170073 mouse PK data

Route	Dose, mg/kg	C_max_, µg/mL	*T*_max_, h	AUC, µg/mL h	*T*_1/2,_ h	CL, L/h	%F
IV	1	0.081	0.25	0.1415	4.4	0.125	
Oral	5	0.049	0.25	0.1237	3.4	0.73	17.5

In vivo pharmacokinetic properties (noncompartmental analysis) of MC-170073 were determined in adult C57BL/6 male mice (2–4 mo old, body weight ranging from 20 to 25 g (Charles River Laboratories, *n* = 3), using a 1 mg/kg intravenous dose and a 5 mg/kg dose via gavage (*n* = 3). Serial blood samples were collected and analyzed at 5, 15, and 30 min, 1, 2, 4, 8, and 24 h after intravenous administration, and at 15, 30 min, 1, 2, 4, 8, and 24 h after gavage administration. AUC, area under the curve; CL, clearance; C_max_, maximal concentration; %F, oral bioavailability; IV, intravenous; PK, pharmacokinetic; *T*_1/2_, half life; T_max_, time to peak drug concentration.

### Administration of MC-170073 Delays Onset and Decreases the Severity of Acute and Chronic DSS-Induced Colitis

**Figure 2.**
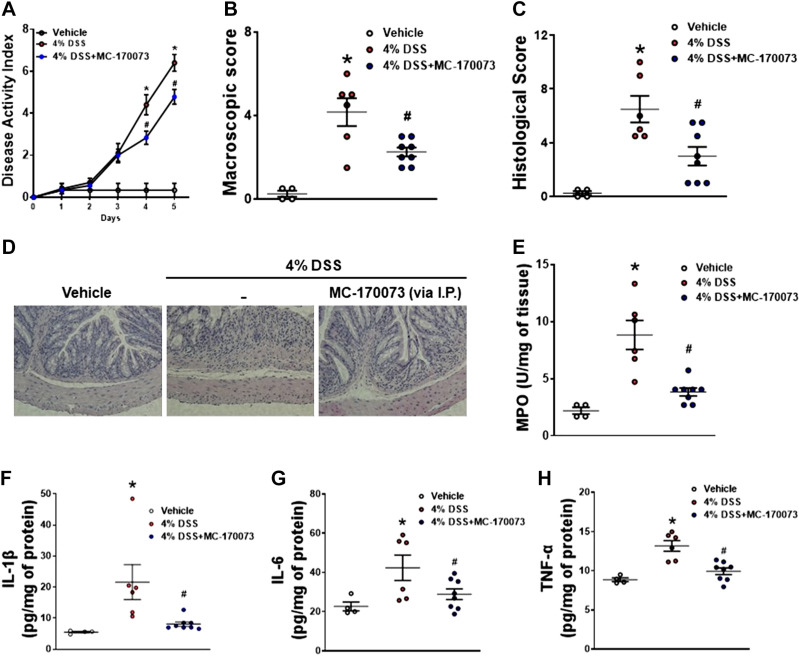
Administration of 5-HT_7_ antagonist (MC-170073) attenuates the severity of acute DSS-induced colitis. In an acute model of colitis, C57BL/6 mice were treated once daily with MC-170073 (10 mg/kg ip) for 6 days starting 1 day before the beginning of the 5-day period of 4% DSS. *A*: disease activity index (DAI). *B*: macroscopic score. *C*: histological score. *D*: representative images of hematoxylin-eosin (H&E)-stained colon sections. *E*: colonic MPO level. *F*: colonic IL-1β levels. *G*: colonic IL-6 levels. *H*: colonic TNF-α levels. Each symbol represents an individual mouse. Data are represented as means ± SE from 4 to 8 mice per group. Statistical significance was determined by one-way analysis of variance (ANOVA) with Bonferroni multiple comparison. *P < 0.05 compared with vehicle. #*P* < 0.05 compared with DSS. DSS, dextran sulfate sodium; MPO, myeloperoxidase; 5-HT_7_, 5-hydroxytryptamine receptor type 7.

**Figure 3.**
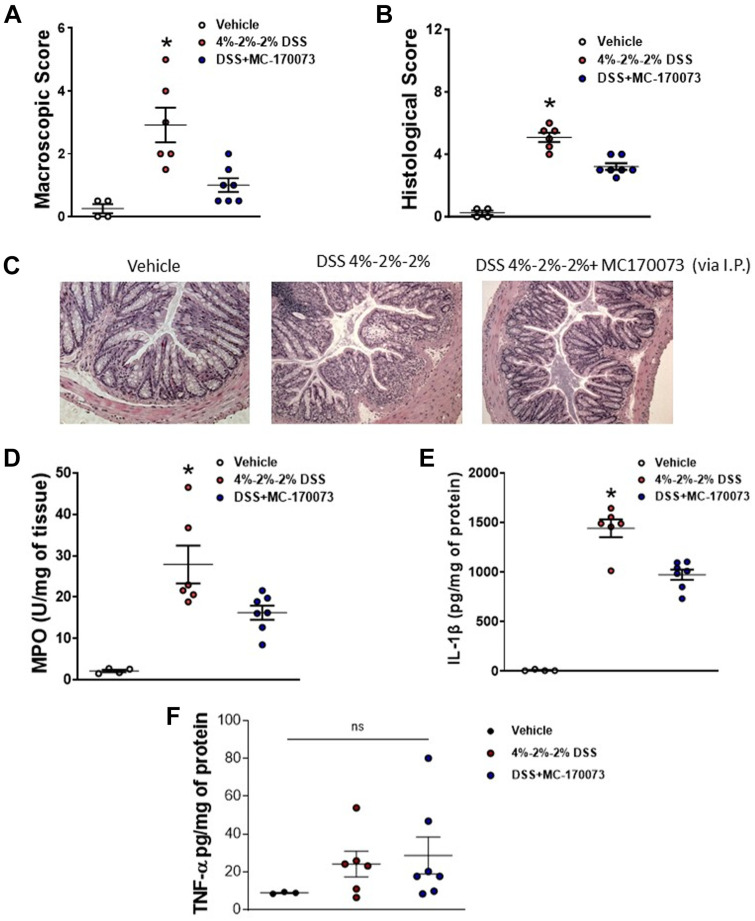
Administration of 5-HT_7_ antagonist (MC-170073) attenuates the severity of chronic DSS-induced colitis. In a chronic model of colitis, C57BL/6 mice were subjected to three cycles of DSS and, during the last cycle of DSS, were treated with MC-170073 (10 mg/kg ip). *A*: macroscopic score. *B*: representative images of hematoxylin-eosin (H&E)-stained colon sections. *C*: histological score. *D*: colonic MPO level. *E*: colonic IL-1β levels.* F:* colonic TNF-α levels. Each symbol represents an individual mouse. Data are represented as means ± SE from 4 to 7 mice per group. Statistical significance was determined by one-way analysis of variance (ANOVA) with Bonferroni multiple comparisons. *P < 0.05 compared with vehicle. DSS, dextran sulfate sodium; MPO, myeloperoxidase; 5-HT_7_, 5-hydroxytryptamine receptor type 7.

Because of the role that 5-HT signaling plays in the pathogenesis of intestinal inflammation, the ability of MC-170073 to attenuate the development of colitis in an acute DSS-induced colitis model was tested. C57BL/6 mice were treated once daily with MC-170073 (10 mg/kg ip) for 6 days, starting 1 day before the beginning of the 5-day period of 4% DSS (Fig. 2). Treatment with MC-170073 resulted in significantly lower DAI scores (Fig. 2*A*) and lower macroscopic scores (Fig. 2*B*) as compared with vehicle-treated mice after induction of colitis with DSS. In addition, histological damage scores were significantly less severe, as illustrated by H&E-stained colon sections that showed less distortion of the epithelium architecture, less goblet cell depletion, and reduced leukocyte infiltration (Fig. 2, *C* and *D*). The reduced severity of colitis in MC-170073-treated mice given DSS was also associated with lower levels of both colonic MPO (Fig. 2*E*) and the proinflammatory cytokines IL-1β, IL-6, and TNF-α (Fig. 2, *F–H*). In a separate experiment, C57BL/6 mice were subjected to three cycles of DSS and, during the last cycle of DSS, were treated with MC-170073 (10 mg/kg) to investigate the effect of this compound in a chronic DSS colitis model (Fig. 3). We observed the attenuation of the severity of colitis in mice treated with MC-170073, as indicated by the downregulation of macroscopic and histological scores along with lower levels of MPO and the proinflammatory cytokine IL-1β (Fig. 3, *A–F*).

### The Single Enantiomer, MC-230078, Alleviates the Severity of DSS-Induced Colitis

**Figure 4.**
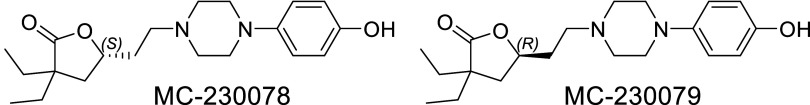
Structure of enantiomers MC-230078 and MC-230079.

The results of the aforementioned DSS studies using MC-170073 were notably similar to previous observations with the antagonist SB-269970 (10). To further expand on this work and to determine if differences between the enantiomers of MC-170073 affected this antagonist’s efficacy in the context of colitis, individual enantiomers of MC-170073 were prepared. It is well known that enantiomers can have different pharmacological properties and that regulatory agencies rarely approve racemic compounds for clinical use. An assessment of the in vivo pharmacokinetic profile of the two enantiomers (Fig. 4 and Table 3) revealed that although the *S*-enantiomer (MC-230078) has a moderate half-life (IV *T*_1/2_ = 3.97 h, PO *T*_1/2 _ = 3.78 h), the half-life of the *R*-enantiomer (MC-230079) was substantially shorter (IV *T*_1/2_ = 0.335 h, PO *T*_1/2 _ = 0.582 h). Further assessment of MC-230078 in 5-HT_7_ assays and off-target 5-HT receptor assays demonstrated that this compound is a potent, selective 5-HT_7_ antagonist (5-HT_7_ *K*_i_ = 106 nm, *K*_b_ = 21 nM, all other 5-HT receptor *K*_i_ >10,000 nM). We also determined that MC-230078 is highly soluble in aqueous media (sol = 200 µM), has limited capacity to inhibit key Cyp enzymes (Cyp3a4 IC_50_ = 9,460 nM; Cyp 2D6 and 2C9m IC_50_s > 10,000 nM), and is highly stable in the presence of human liver microsomes (HLM *T*_1/2_ = 60 min). Based on these data, we elected to execute additional in vivo efficacy studies on MC-230078.

**Table 3.** In vivo mouse PK of enantiomer MC-230078 and MC-230079

Route	Dose, mg/kg	C_max_, µg/mL	*T*_max_, h	AUC, µg/mL h	*T*_1/2_, h	CL, L/h	%F
*S-enantiomer (MC-230078)*							
IV	1	0.527	0.083	0.277	3.97	0.084	
Oral	5	0.123	0.5	0.19	3.78	0.511	13.7
*R-enantiomer (MC-230079)*							
IV	1	0.148	0.167	0.097	0.335	0.252	
Oral	5	0.057	0.5	0.0525	0.582	2.07	10.8

In vivo pharmacokinetic properties (noncompartmental analysis) of MC-230078 and MC-230079 were determined in adult C57BL/6 male mice (2–4 mo old, body weight ranging from 20 to 25 g, Charles River Laboratories, *n* = 3) using a 1 mg/kg intravenous dose and a 5 mg/kg dose via oral gavage (*n* = 3). Serial blood samples were collected and analyzed at 5, 15, and 30 min, 1, 2, 4, 8, and 24 h after intravenous administration, and at 15, 30 min, 1, 2, 4, 8, and 24 h after gavage administration. AUC, area under the curve; CL, clearance; C_max_, maximal concentration; %F, oral bioavailability; IV, intravenous; PK, pharmacokinetic; T_1/2_, half life; T_max_, time to peak drug concentration.

**Figure 5.**
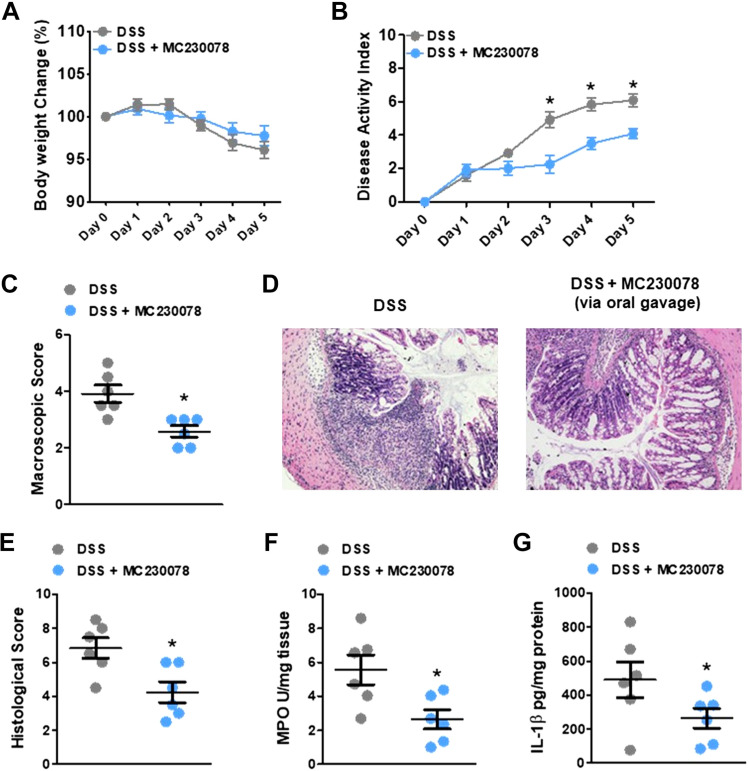
Administration of 5-HT_7_ antagonist (MC-230078) attenuates the severity of DSS-induced colitis. MC-230078 at 10 mg·kg^−1^ body weight was orally administered twice daily starting from *day 0* of 5% DSS for 5 days. *A*: body weight change. *B*: disease activity index (DAI). *C*: macroscopic score. *D*: representative images of hematoxylin-eosin (H&E)-stained colon sections. *E*: histological score. *F*: colonic MPO level. *G*: colonic IL-1β level. Each symbol represents an individual mouse. Data are represented as means ± SE from 6 mice per group. Statistical significance was determined by unpaired Student’s *t* test. *P < 0.05 compared with DSS. DSS, dextran sulfate sodium; MPO, myeloperoxidase; 5-HT_7_, 5-hydroxytryptamine receptor type 7.

To assess the effects of treatment with MC-230078 on intestinal inflammation, colitis was induced with 5% DSS in drinking water for 5 days in C57BL/6 mice, and starting from *day 0*, MC-230078 at 10 mg/kg was administered orally twice daily for 5 days (Fig. 5). There was no significant difference in the body weight change during DSS (Fig. 5*A*); however, MC-230078 treatment resulted in significantly lower DAI (Fig. 5*B*). We also observed significantly lower macroscopic scores in mice treated with MC-230078 as compared with vehicle-treated mice on *day 5* post-DSS (Fig. 5*C*). H&E-stained colon cross-sections of mice treated with MC-230078 showed significantly lower histological scores compared with vehicle-treated mice on *day 5* post-DSS (Fig. 5, *D* and *E*). In addition, a reduction in colitis severity was associated with significantly lower colonic MPO and IL-1β levels (Fig. 5*, F* and *G*). Similar effects were found with the administration of 4% DSS for 5 days (see Supplemental Fig. S1). It should also be noted that administration of either MC-170073 or MC-230078 in the absence of DSS had no significant effect on body weight change, histology, or proinflammatory cytokine production (see Supplemental Fig. S2).

### MC-230078 Attenuates the Severity of CD4^+^CD45RB^high^ T Cell-Induced Colitis

**Figure 6.**
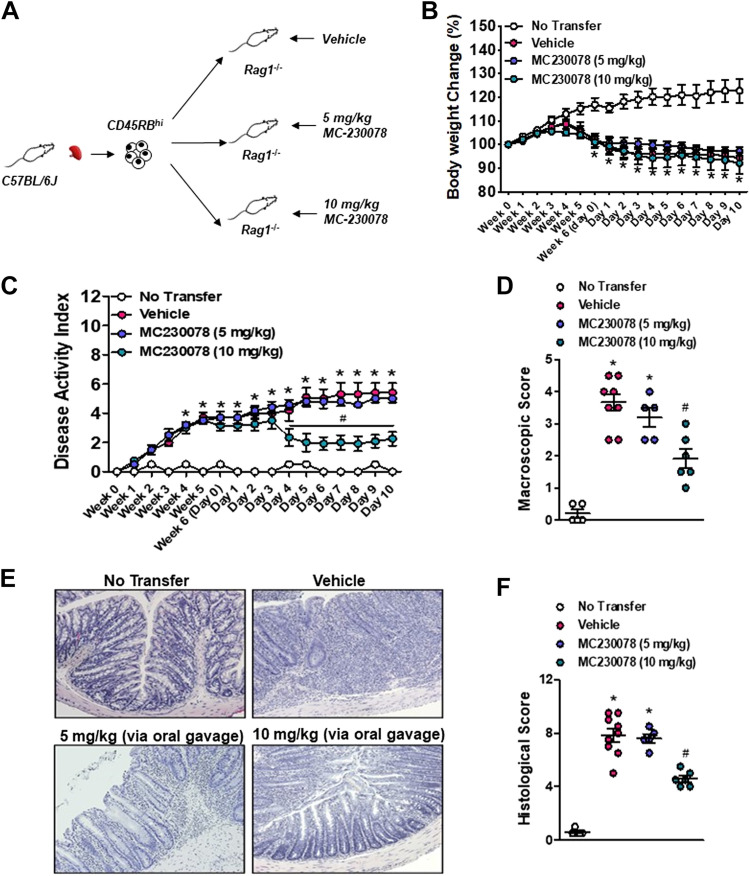
Administration of 10 mg/kg of 5-HT_7_ antagonist (MC-230078) alleviates the development of CD45RB^high^ T cell-induced colitis. *A*: experimental design. Briefly, *Rag1*^−/−^ (C57BL/6J background) mice were reconstituted with FACS-sorted 5.0 × 10^5^ CD4^+^CD45RB^hi^ T cells harvested from the spleen of healthy C57BL/6J mice. At *week 6*, either vehicle or MC-230078 (5 or 10 mg·kg^−1^ body wt) was orally administered twice daily for 10 days before euthanization. *B*: body weight change. *C*: disease activity index (DAI). *D*: macroscopic score. *E*: representative images of hematoxylin-eosin (H&E)-stained colon sections. *F*: histological score. Each symbol represents an individual mouse. Data are represented as means ± SE from 5 to 9 mice per group. *P < 0.05 compared with no transfer. #*P* < 0.05, compared with vehicle. Statistical significance was determined by unpaired Student’s *t* test, one-way or two-way analysis of variance (ANOVA) with Bonferroni multiple comparisons. 5-HT_7_, 5-hydroxytryptamine receptor type 7.

**Figure 7.**
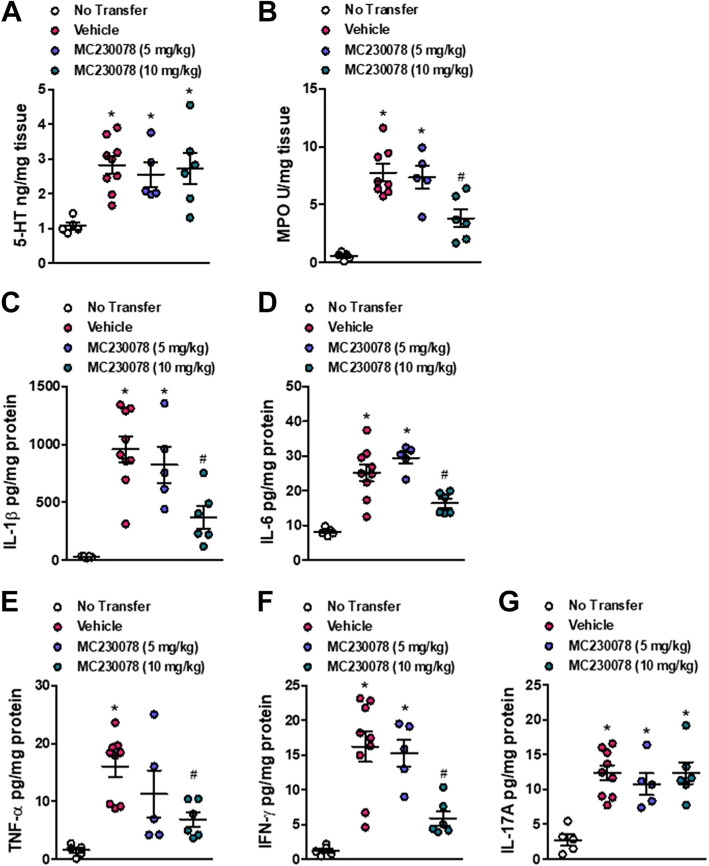
5-HT_7_ antagonist (MC-230078) at 10 mg/kg reduces the production of MPO and proinflammatory cytokines in a CD45RB^high^ T cell-induced colitis. *A*: colonic 5-HT level. *B*: colonic MPO level. *C–G*: levels of colon proinflammatory cytokines (IL-1β, IL-6, TNF-α, IFN-γ, and IL-17A). Each symbol represents an individual mouse. Data are represented as means ± SE from 5 to 9 mice per group. Statistical significance was determined by one-way analysis of variance (ANOVA) with Bonferroni multiple comparison. *P < 0.05 compared with no transfer. #*P* < 0.05 compared with vehicle. MPO, myeloperoxidase; 5-HT_7_; 5-hydroxytryptamine receptor type 7.

To further investigate the effect of MC-230078 in the context of intestinal inflammation, the effects of this compound were studied in the CD4^+^CD45RB^high^ T cell-induced chronic colitis model, one of the best-characterized models of colitis that closely resembles human IBD. Briefly, FACS-sorted wild-type (WT) CD4^+^CD45RB^high^ T cells were intraperitoneally reconstituted into *Rag1*^−/−^ mice. At *week 6* post-reconstitution, mice were orally administered MC-230078 (5 or 10 mg/kg) twice daily for 10 days (Fig. 6*A*). Body weight change was significantly different between *Rag1*^−/−^ mice without T cell transfer and *Rag1*^−/−^ with T cell reconstitution (vehicle), whereas there was no difference between the two drug-treated groups in terms of body weight change (Fig. 6*B*). Starting 4 days into the drug administration period, mice treated at 10 mg/kg, but not 5 mg/kg, showed a significant reduction in DAI compared with vehicle-treated mice (Fig. 6*C*). There was also reduced macroscopic damage in mice treated with 10 mg/kg of MC-230078 (Fig. 6*D*), compared with vehicle-treated mice. In addition, histological damage scores were significantly lower when mice were treated with a higher dosage of MC-230078 (Fig. *6, E* and *F*). Further assessment of the effect of MC-230078 on inflammation was done by measuring the levels of colonic MPO, colonic 5-HT, and proinflammatory cytokines in the colon. All mice receiving the T cell transfer exhibited higher colonic 5-HT levels compared with those mice that did not receive T cell reconstitution (Fig. 7*A*). Treatment with MC-230078 at 10 mg/kg ameliorated T cell-induced colitis, and this reduction in colitis severity was associated with a significant decrease in colonic MPO levels (Fig. 7*B*) and a significant downregulation of the proinflammatory cytokines, IL-1β, IL-6, TNF-α, and IFN-γ compared with vehicle-treated mice (Fig. 7, *C–G*).

## DISCUSSION

Approximately one-third of patients with IBD do not respond appropriately to existing biologics or immunosuppressive agents, effects that may ultimately lead to surgical interventions to minimize symptomatology and disease progression. In experimental models of both DSS and DNBS colitis, our group has previously reported the beneficial effects of pharmacologically blocking 5-HT_7_ receptors via the selective antagonist SB-269970, which in the colons of C57BL/6 mice resulted in a reduced immune response and intestinal inflammation (10). Here, we explored the role of the novel selective 5-HT_7_ antagonists, MC-170073 and MC-230078, in intestinal inflammation by using two different experimental models of colitis.

Previous work from our group has shown that intestinal inflammation in both experimental models and patients with IBD is associated with increased 5-HT signaling and 5-HT_7_ receptor expression (10, 13, 20). Studies using animal models of colitis have also linked intestinal inflammation to the dysregulation of 5-HT signaling. Specifically, mice lacking the rate-limiting enzyme for biosynthesis of 5-HT, tryptophan hydroxylase 1 (TPH1), showed significantly reduced severity of intestinal inflammation in both DSS and DNBS models of colitis (21). Furthermore, 5-HT_7_^−/−^ mice exhibited significantly lower severity of intestinal inflammation compared with wild-type mice (10). Given that DSS alters the availability of colonic 5-HT (21, 22) and increases the expression of 5-HT_7_ receptors in the colon (10), pharmacological inhibition of these receptors may prevent a positive feedback loop, which promotes the dysregulated inflammatory environment common in IBD. It should be noted that in contrast to our previous work, Guseva et al. (23) reported that antagonizing 5-HT_7_ with SB-269970 or eliminating 5-HT_7_ via genetic manipulation leads to increased inflammatory responses and lethality upon exposure to DSS; DSS exposure at 4% wt/vol resulted in the death of 33% of the untreated animals, whereas mice treated with SB-269970 had a mortality rate of >60%. Key differences, including the dosing of DSS, the dosing of antagonists, and differences in housing conditions, may account for these discrepancies. Potential off-target effects specifically with regard to 5-HT_1B_, 5-HT_5A_, and D_2_ activity, as suggested by Guseva et al. (23), may have also been a concern at variable doses; however, data from the PDSP *K*_i_ database (https://pdsp.unc.edu/databases/pdsp.php) demonstrate that SB-269970 is highly selective for 5-HT_7_ over the aforementioned receptors (>100-fold selective). In our studies, DSS exposure did not produce lethality, nor did exposure to SB-269970, despite the use of a substantially higher dose. It should also be noted that DSS exposure is not known to increase mortality when properly optimized (24).

Antagonizing 5-HT_7_ receptors may also open up roles for targeting other 5-HT receptors in the intestinal mucosa. Among the seven types of 5-HT receptors, 5-HT_1_, 5-HT_2_, 5-HT_3_, 5-HT_4_, and 5-HT_7_ are expressed within the GI tract, with 5-HT_3_ and 5-HT_4_ receptors being the most widely studied. Prior investigations established that DSS decreases the expression of 5-HT_4_ receptors (25), whereas the activation of this receptor with an agonist (tegaserod) exerted protective actions on the development of colitis induced by DSS and TNBS (26). The role of 5-HT_3_ receptors has also been evaluated in experimental models of colitis. However, both pro- and anti-inflammatory effects have been observed (27, 28). Recently, we have also shown that increased 5-HT levels in the gut impair autophagy and increase the severity of colitis. By contrast, targeted inhibition of 5-HT signaling promotes autophagy, which subsequently modulates the gut microbiota and reduces the severity of colitis (29). Autophagy, a process of cellular recycling, has been implicated in the pathogenesis of various diseases, including IBD. Though 5-HT can impair autophagy via 5-HT_3_, 5-HT_4_, and 5-HT_7_ receptors expressed on intestinal epithelial cells (IECs), 5-HT_7_ receptors appeared to play a major role. These findings further suggest a key role of 5-HT_7_ in the regulation of inflammatory processes in the context of colitis. Together, these findings suggest that 5-HT exerts pleiotropic receptor-dependent effects to influence inflammation and plays a key role as a signaling molecule in colitis. In conjunction with this, the data presented herein suggest that the proinflammatory effect of 5-HT signaling can be ameliorated by targeted pharmacological inhibition of 5-HT_7_ receptors.

It should be noted that in addition to IECs, T lymphocytes are one of several subsets of immune cells that express 5-HT receptors and are functionally responsive to 5-HT (30, 31). Intriguingly, naive CD4^+^ T cells expressing 5-HT_7_ receptors can induce rapid T cell responses upon 5-HT exposure ex vivo, a response that was abrogated by the antagonist SB-269970 (32). Colonic inflammation in the CD4^+^CD45RB^high^ T cell transfer model of colitis is governed by the antigen-driven activation, polarization, and expansion of naive T cells to colitogenic effector cells, such as Th1 and/or Th17 cells, and subsequently elevated and unchecked production of proinflammatory cytokines (33, 34). In mice reconstituted with naive CD45RB^high^ T cells, increased severity of intestinal inflammation is associated with higher colonic 5-HT levels, a state attributed to the ability of 5-HT, acting as an intrinsic cofactor, to promote the activation and enhance the responses of naive CD4^+^ T cells (32). In the CD45RB^high^ T cell-induced colitis model, no differences in colonic 5-HT levels in mice treated with MC-230078 were observed when compared with vehicle-treated mice. These findings suggest that even if colonic 5-HT levels are increased, pharmacological inhibition of 5-HT_7_ receptors can efficiently ameliorate intestinal inflammation, as seen by reduced histopathological damage and diminished levels of proinflammatory cytokines. 5-HT_7_ receptors expressed by dendritic cells (DCs) also play a pivotal role in the 5-HT-mediated proinflammatory response (10, 35). 5-HT influences cytokine secretion by DCs and primes T cells for the production of Th1 and Th17 cytokines (35). Numerous studies have shown that Th1 and Th17 subsets of CD4^+^ T cells play distinct roles in the pathogenesis of IBD (33, 36, 37). Both Th1- and Th17-type immune responses have also been characterized in animal models of colitis, such as DSS-induced and CD45RB^high^ T cell-induced colitis (18, 37, 38). It should be noted that the expression of IFN-γ, a Th1 cytokine, by T cells is critical for T cell-induced colitis (39), and IFN-γ has served as an effective target for antibody-based therapies in IBD trials (40). Thus, by targeting 5-HT_7_ receptors and influencing both DC and T cell functionality and inflammatory potential, MC-230078 may provide an alternative treatment strategy for those suffering from IBD. Future studies exploring the pharmacological inhibition of 5-HT_7_ receptors by MC-230078 and its effect on the transition of precursors to Th1-like and Th17-like cells are warranted. Previously, in studies done with radiation chimera employing bone marrow cells (BMCs) from 5-HT_7_ receptor-deficient (5-HT_7_^−/−^) mice, we have shown that 5-HT plays a key role in the activation of immune cells via the 5-HT_7_ receptor in relation to colitis pathogenesis (10). We also observed that CD11c+ DCs isolated from mice given BMCs from 5-HT_7_^−/−^ mice produced significantly lower levels of IL-1β and IL-6 in the presence of lipopolysaccharide (LPS) when compared with CD11c+ DCs isolated from controls. In addition, 5-HT upregulated cytokine production by LPS-matured DCs through activation of the NF-κB pathway, and this was mediated by the 5-HT_7_ receptor. Taken together, these observations suggest that these 5-HT_7_ antagonists may modulate the severity of inflammation by inhibiting 5-HT_7_ receptor-mediated proinflammatory mediator production from DCs.

In conclusion, the newly identified 5-HT_7_ receptor antagonists, namely, MC-170073 and MC-230078, which express high binding potencies and functional efficacy, aid in the amelioration of intestinal inflammation in both DSS-induced and CD45RB^high^ T cell-induced models of colitis. This work provides further support that the administration of selective 5-HT_7_ receptor antagonists may be beneficial in the context of intestinal inflammation, and in the future, translation of these findings may provide a new therapeutic strategy for treating patients with IBD.

## DATA AVAILABILITY

Data will be made available upon request to the corresponding author.

## SUPPLEMENTAL DATA

10.17632/vwycbkk9x2.1 Supplemental Figs. S1 and S2 and Supplemental Table S1: https://doi.org/10.17632/vwycbkk9x2.1.

## GRANTS

This work was supported by grants from Canadian Institutes of Health Research Grant PJT 156262 (to W.I.K.) and Crohn’s & Colitis Foundation Grant 510490 (to B.E.B. and D.C.).

## DISCLOSURES

No conflicts of interest, financial or otherwise, are declared by the authors.

## AUTHOR CONTRIBUTIONS

Y.H.K., B.E.B., D.J.C., and W.I.K. conceived and designed research; Y.H.K., H.W., J.A.G., S.B., K.K., M.Y., J.C.G., D.C., and K.M.B. performed experiments; Y.H.K., B.E.B., H.W., D.J.C., and W.I.K. analyzed data; Y.H.K., B.E.B., D.J.C., and W.I.K. interpreted results of experiments; Y.H.K. prepared figures; Y.H.K., B.E.B., J.A.G., D.J.C., and W.I.K. drafted manuscript; B.E.B, J.A.G., D.J.C., and W.I.K. edited and revised manuscript; Y.H. K., B.E.B., H.W., J.A.G., D.J.C., and W.I.K. approved final version of manuscript.
